# Mid- to Long-Term Clinical and Echocardiographic Effects of Post-procedural Permanent Pacemaker Implantation After Transcatheter Aortic Valve Replacement: A Systematic Review and Meta-Analysis

**DOI:** 10.3389/fcvm.2022.911234

**Published:** 2022-06-28

**Authors:** Shun Xu, Enrui Zhang, Zhiyong Qian, Jinyu Sun, Fengwei Zou, Yao Wang, Xiaofeng Hou, Jiangang Zou

**Affiliations:** ^1^Department of Cardiology, The First Affiliated Hospital of Nanjing Medical University, Nanjing, China; ^2^Montefiore Medical Center, Bronx, NY, United States

**Keywords:** transcatheter aortic valve replacement, permanent pacemaker implantation, mortality, heart failure rehospitalization, left ventricular ejection fraction, meta-analysis

## Abstract

**Aims:**

To date, the prognostic effects of permanent pacemaker implantation (PPI) after transcatheter aortic valve replacement (TAVR) remain controversial. The purpose of this meta-analysis was to investigate the mid- (1 year) to long-term (> 1 year) clinical and echocardiographic effects of post-procedural PPI in patients after TAVR.

**Methods:**

PubMed, Embase, Web of Science, and Cochrane Library databases were systematically searched from the establishment of databases up to 1 December 2021. Studies comparing clinical and echocardiographic outcomes between patients with and without post-TAVR PPI of ≥ 1-year follow-up were collected for further meta-analysis.

**Results:**

A total of 39 studies comprising of 83,082 patients were included in this meta-analysis. At mid-term follow-up (1 year), the pooled results demonstrated a higher risk of all-cause mortality in patients with post-procedural PPI than those without following TAVR (relative risk (RR), 1.17; 95% CI, 1.10–1.24; *P* < 0.00001). No significant differences were observed in cardiovascular mortality (RR, 0.86; 95% CI, 0.71–1.03; *P* = 0.10) or heart failure rehospitalization (RR, 0.91; 95% CI, 0.58–1.44; *P* = 0.69) at 1-year follow-up. At long-term follow-up (> 1 year), post-TAVR PPI had negative effects on all-cause mortality (RR, 1.18; 95% CI, 1.09–1.28; *P* < 0.0001) and heart failure rehospitalization (RR, 1.42; 95% CI, 1.18–1.71; *P* = 0.0002). There was no difference in long-term cardiovascular mortality between the two groups (RR, 1.15; 95% CI, 0.97–1.36; *P* = 0.11). Left ventricular ejection fraction (LVEF) was not significantly different at baseline (mean difference, 1.40; 95% CI, –0.13–2.93; *P* = 0.07), but was significantly lower in the PPI group at 1-year follow-up (mean difference, –3.57; 95% CI, –4.88 to –2.26; *P* < 0.00001).

**Conclusion:**

Our meta-analysis provides evidence that post-TAVR PPI has negative clinical and echocardiographic effects on patients at mid- to long-term follow-up. Further studies are urgently needed to explore the cause of these complications and optimize the treatment and management of patients requiring permanent pacing after TAVR.

**Systematic Review Registration:**

[https://www.crd.york.ac.uk/prospero/display_record.php?ID=CRD42021289935], identifier [CRD42021289935].

## Introduction

Transcatheter aortic valve replacement (TAVR) has become a well-established therapy for patients with severe aortic stenosis and high risk for surgical aortic valve replacement (1, 2). Recent randomized controlled trials provided evidence to extend the application of TAVR to low-risk patients (3, 4). Despite technological advances and clinical experience accumulation, atrioventricular node, and infranodal tissues remain easily impaired during the implantation of the valve prosthesis. Conduction abnormalities (e.g., high-degree atrioventricular block and new-onset persistent left bundle branch block) are frequently observed after TAVR, and patients often require permanent pacemaker implantation (PPI) (5). The application of post-TAVR PPI was reported in approximately 2.3–37.7% of patients, and the rates largely vary according to the types and generations of the transcatheter valves (6).

Cardiac pacing is a recommended therapy to reduce the risk of death related to severe bradycardia arrhythmias. However, traditional right ventricular pacing (RVP) can cause electrical and mechanical dyssynchrony (7, 8), thus increasing the risk of mortality and heart failure hospitalization (9–11). Currently, it remains controversial whether the application of PPI could influence the clinical symptoms and survival outcomes after TAVR (12). Previous meta-analyses were limited by a small number of studies or lack of long-term follow-up (13, 14). This meta-analysis aims to investigate the mid- to long-term clinical and echocardiographic outcomes of post-procedural PPI in patients after TAVR.

## Methods

### Search Strategy

We performed a systematic literature search in PubMed, Embase, Web of Science, and Cochrane Library from the establishment of databases up to 1 December 2021 by two investigators (Shun Xu and Enrui Zhang) independently. The following strategy was used in PubMed: ((((((“Transcatheter Aortic Valve Replacement” [Mesh]) OR (Transcatheter Aortic Valve Replacement [Title/Abstract])) OR (Transcatheter Aortic Valve Implantation [Title/Abstract])) OR (TAVR [Title/Abstract])) OR (TAVR [Title/Abstract]))) AND (((((“Cardiac Pacing, Artificial” [Mesh]) OR (pacing [Title/Abstract])) OR (pace [Title/Abstract])) OR ((“Pacemaker, Artificial” [Mesh]) OR (pacemaker [Title/Abstract])))). The searching strategies for Embase, Web of Science, and Cochrane Library were provided in Supplementary Table 1. We also manually screened reference lists of retrieved reviews, reports, and other relevant publications to identify additional pertinent studies.

### Study Design

The protocol of this meta-analysis has been registered in PROSPERO (Registration ID: CRD42021289935). Clinical studies were eligible if they met the following inclusion criteria: (1) studies comparing clinical and echocardiographic outcomes between patients with and without post-procedural PPI after TAVR, including all-cause mortality, cardiovascular mortality, heart failure rehospitalization, and left ventricular ejection fraction (LVEF); (2) studies with a follow-up of ≥ 1 year; (3) studies with full texts published in English in peer-reviewed journals. We only included the study containing the most data for multiple publications of the same trial. We excluded review articles, case reports, letters, editorials, articles lacking outcomes of interest, studies without detailed data, and studies with a follow-up of < 1 year. Importantly, we also excluded studies that failed to distinguish patients with PPI before TAVR. Two independent investigators (Shun Xu and Enrui Zhang) assess eligibility by screening and reviewing article titles, abstracts, and full texts. Any disagreement about eligibility was clarified *via* consulting a third investigator (Jinyu Sun).

### Data Extraction and Quality Assessment

Two investigators (Shun Xu and Enrui Zhang) independently extracted data for each eligible study. Any disagreement was resolved through discussion with a third investigator (Jinyu Sun) to reach a consensus. The following characteristics were included: first author, year of publication, inclusion period, number and region of centers, sample size, PPI criteria, patient demographic characteristics, and the following mid-term (1 year) to long-term (> 1 year) outcomes, including all-cause mortality, cardiovascular mortality, heart failure rehospitalization, and LVEF.

The quality of studies involved was assessed by two investigators (Shun Xu and Enrui Zhang) independently using the Newcastle-Ottawa Scale (NOS). The NOS tool involved three aspects, and a maximum of 9 stars can be allotted to each study: the selection of cohorts (0–4 stars), the comparability of cohorts (0–2 stars), and the assessment of the outcome (0–3 stars). A NOS score ≥ 6 stars indicated moderate-to-high quality, while a NOS score < 6 stars indicated low quality. Discrepancies were resolved by consulting a third investigator (Jinyu Sun) to reach a consensus.

### Statistical Analysis

Continuous variables were presented as mean ± standard deviation, and categorical variables were presented as frequencies or percentages. Relative risk (RR) with corresponding 95% confidence intervals (CIs) for each endpoint was calculated and analyzed for categorical variable outcomes. Continuous data were summarized as a mean difference with 95% CI. *P*-value < 0.05 was considered statistically significant. The heterogeneity between studies was quantified by *I*-squared (*I*^2^) statistic, with a fixed-effects model adopted when the *I*^2^-value was < 50% and a random-effects model applied otherwise. Review Manager version 5.3 was used for all the statistical analyses. The meta-analysis was performed according to the Preferred Reporting Items for Systematic Reviews and Meta-Analyses (PRISMA) guidelines (15).

## Results

### Study Selection and Quality Assessment

**FIGURE 1:**
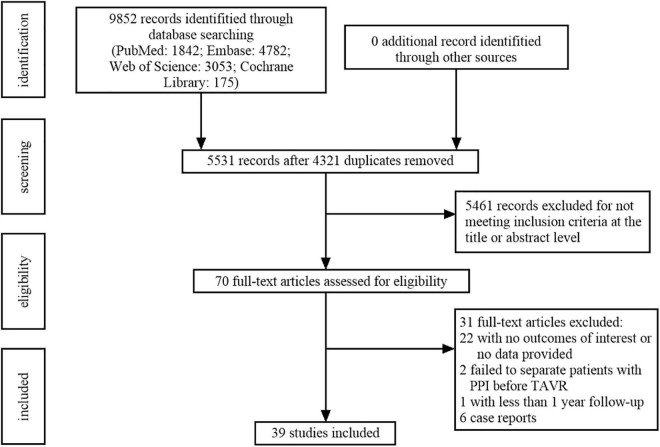
Flowchart of study selection based on the Preferred Reported Items for Systematic Reviews and Meta-Analysis (PRISMA) statement.

Figure 1 shows the flow chart of the study selection. A total of 9,852 records were initially identified from the databases according to the searching strategies, including 1,842 from PubMed, 4,782 from Embase, 3,053 from Web of Science, and 175 from Cochrane Library. After title and abstract screening, a total of 4,321 duplicates and 5,461 irrelevant records were excluded, the remaining 70 full-text articles to be reviewed for eligibility. Of those, 22 studies were excluded for having no outcomes of interest or without provided data. Two studies were excluded due to failing to distinguish patients with PPI before TAVR. One study was excluded because the follow-up duration was less than 1 year. Six case reports were also excluded. Finally, 39 studies containing 83,082 patients were included for further analysis (16–54) (Table 1).

**TABLE 1:** Summary of studies evaluating mid- to long-term clinical and echocardiographic effects of post-TAVR PPI.

References	Year	Region	Centers	Inclusion period	Sample	PPI criteria	Time of PPI
Rück et al. (16)	2021	Sweden	8	Jan 2008–Dec 2018	3420	NA	≤30 days
Rajah et al. (17)	2021	Arabia	1	Jan 2010–Jan 2019	170	NA	≤30 days
Schoechlin et al. (18)	2021	Germany	1	Jan 2014–Dec 2016	767	Restrictive or liberal strategy	After TAVR
Van Mieghem et al. (19)	2021	International	53	Jan 2016–Dec 2016	886	NA	≤30 days
Clementy et al. (20)	2021	France	NA	Jan 2010–Jun 2019	23060	NA	≤30 days
Weferling et al. (21)	2021	Germany	1	Jan 2010–Apr 2019	1846	ESC 2013 guidelines	Median 3 days
Nicolas et al. (22)	2021	Europe and United States	19	Jan 2013–Dec 2015	922	ESC 2013 guidelines	After TAVR
Alperi et al. (23)	2021	International	>180	Apr 2007–Apr 2020	1987	NA	Before discharge
Ashraf et al. (24)	2020	United States	1	Jan 2012–Jul 2018	243	ACC/AHA/HRS guidelines	≤30 days
Duet al. (25)	2020	China	1	Mar 2013–Oct 2018	256	ACC/AHA/HRS 2012 guidelines	≤30 days
Fujita et al. (26)	2020	Germany	NA	2011–2015	20872	NA	Before discharge
Costa et al. (27)	2019	Italy	1	Jun 2007–Feb 2018	1116	ESC 2013 guidelines	≤30 days
Meduri et al. (28)	2019	International	55	Sep 2014–Dec 2015	688	NA	≤30 days
Maeno et al. (29)	2019	United States	1	Jan 2013–Dec 2015	659	NA	Before discharge
Jørgensen et al. (30)	2019	Denmark	1	Aug 2007–Sep 2017	816	NA	≤30 days
Gonska et al. (31)	2018	Germany	1	Feb 2014–Sep 2016	532	NA	After TAVR
Nadeem et al. (32)	2018	United States	1	2011–2017	672	NA	After TAVR
Alasti et al. (33)	2018	Australia	1	Apr 2012–Oct 2016	152	High-degree AVB, first-degree AVB with LBBB, AF with slow ventricular rate and SSS	≤30 days
Walther et al. (34)	2018	International	12	Dec 2011–Sep 2015	198	NA	≤1 year
Rogers et al. (35)	2018	United States	1	Jan 2013–Dec 2015	614	NA	After TAVR
Aljabbary et al. (36)	2018	Canada	10	Apr 2010–Mar 2015	1257	NA	Before discharge
Chamandi et al. (37)	2018	International	9	May 2007–Feb 2015	1629	ACC/AHA/HRS 2012 guidelines	≤30 days
López-Aguilera et al. (38)	2018	Spain	1	Apr 2008–Dec 2015	217	Third-degree AVB, LBBB or new first-degree AVB with persistent severe bradycardia (< 40 bpm) and developed syncope	After TAVR
Nijenhuis et al. (39)	2017	Netherlands	1	Jun 2007–Jun 2015	155	ESC 2007/2013 guidelines	8 (6–14) days
Engborg et al. (40)	2017	Denmark	1	Mar 2008–Sep 2012	128	High-degree AVB, SSS, LBBB combined with first-degree AVB	≤30 days
Fadahunsi et al. (41)	2016	United States	229	Nov 2011–Sep 2014	9785	NA	≤30 days
Giustino et al. (42)	2016	International	4	Nov 2005–Dec 2011	947	ACC/AHA/HRS 2012 guidelines	After TAVR
Dizon et al. (43)	2015	International	25	May 2007–Aug 2009	1945	NA	≤30 days
Mouillet et al. (44)	2015	France	29	Jan 2010–Oct 2011	833	NA	After TAVR
Kawaguchi et al. (45)	2015	France	1	Feb 2010–Jun 2012	160	NA	After TAVR
Schymik et al. (46)	2015	Germany	1	May 2008–Apr 2012	634	ESC 2013 guidelines	After TAVR
Nazif et al. (47)	2015	International	25	NA	1973	High-degree AVB, SSS, and other bradycardias	≤30 days
Urena et al. (48)	2014	International	8	Jan 2005–Feb 2013	1556	ACC/AHA/HRS 2008 guidelines	≤30 days
Biner et al. (49)	2014	Israel	1	NA	230	Pre-TAVR RBBB, post-TAVR high-degree AVB, alternating BBB, and new LBBB with PR-interval prolongation ≥ 280 ms	After TAVR
Pereira et al. (50)	2013	Portugal	1	Aug 2007–May 2011	58	ESC 2007 guidelines	Range 1–9 days
Houthuizen et al. (51)	2012	Netherlands	8	Nov 2005–Dec 2010	797	NA	After TAVR
De Carlo et al. (52)	2012	Italy	3	Sep 2007–Jul 2010	275	ESC 2007 guidelines	Range 0–12 days
Buellesfeld et al. (53)	2012	Switzerland and Germany	2	Aug 2007–Mar 2010	305	High-degree AVB, new LBBB with PR interval prolongation ≥ 300 ms, and AF with inadequate escape rhythm	≤30 days
D’Ancona et al. (54)	2011	Germany	1	Apr 2008–Mar 2011	322	High-degree AVB and symptomatic bradycardia	≤30 days

*TAVR, transcatheter aortic valve replacement; PPI, permanent pacemaker implantation; NA, not available; ESC, European Society of Cardiology; ACC, American College of Cardiology; AHA, American Heart Association; HRS, Heart Rhythm Society; AVB, atrioventricular block; SSS, sick sinus syndrome; AF, atrial fibrillation; LBBB, left bundle branch block; RBBB, right bundle branch block; BBB, bundle branch block.*

All included studies had moderate-to-high quality while none had less than 6 points according to NOS: two with 9 points, nineteen with 8 points, six with 7 points, and 12 with 6 points. The details of the quality assessment are shown in Table 2.

**TABLE 2:** Quality assessment of the included studies according to the Newcastle-Ottawa Scale (NOS).

References	Selection	Comparability	Outcome	Total stars
Rück et al. (16)	4	2	2	8
Rajah et al. (17)	4	2	2	8
Schoechlin et al. (18)	4	0	2	6
Van Mieghem et al. (19)	4	0	2	6
Clementy et al. (20)	4	0	2	6
Weferling et al. (21)	4	0	3	7
Nicolas et al. (22)	4	2	2	8
Alperi et al. (23)	4	1	2	7
Ashraf et al. (24)	4	0	2	6
Du et al. (25)	4	2	2	8
Fujita et al. (26)	4	0	2	6
Costa et al. (27)	4	2	2	8
Meduri et al. (28)	4	2	2	8
Maeno et al. (29)	4	2	2	8
Jørgensen et al. (30)	4	0	2	6
Gonska et al. (31)	4	2	3	9
Nadeem et al. (32)	4	2	2	8
Alasti et al. (33)	4	2	2	8
Walther et al. (34)	4	1	2	7
Rogers et al. (35)	4	0	2	6
Aljabbary et al. (36)	4	0	2	6
Chamandi et al. (37)	4	1	2	7
López-Aguilera et al. (38)	4	2	2	8
Nijenhuis et al. (39)	4	2	2	8
Engborg et al. (40)	4	2	2	8
Fadahunsi et al. (41)	4	1	2	7
Giustino et al. (42)	4	1	2	7
Dizon et al. (43)	4	0	2	6
Mouillet et al. (44)	4	2	2	8
Kawaguchi et al. (45)	4	2	2	8
Schymik et al. (46)	4	0	2	6
Nazif et al. (47)	4	2	2	8
Urena et al. (48)	4	2	2	8
Biner et al. (49)	4	2	2	8
Pereira et al. (50)	4	0	2	6
Houthuizen et al. (51)	4	0	2	6
De Carlo et al. (52)	4	2	2	8
Buellesfeld et al. (53)	4	2	3	9
D’Ancona et al. (54)	4	2	2	8

### Mid-Term (1 Year) Clinical Effects of Post-procedural Permanent Pacemaker Implantation After Transcatheter Aortic Valve Replacement

**FIGURE 2:**
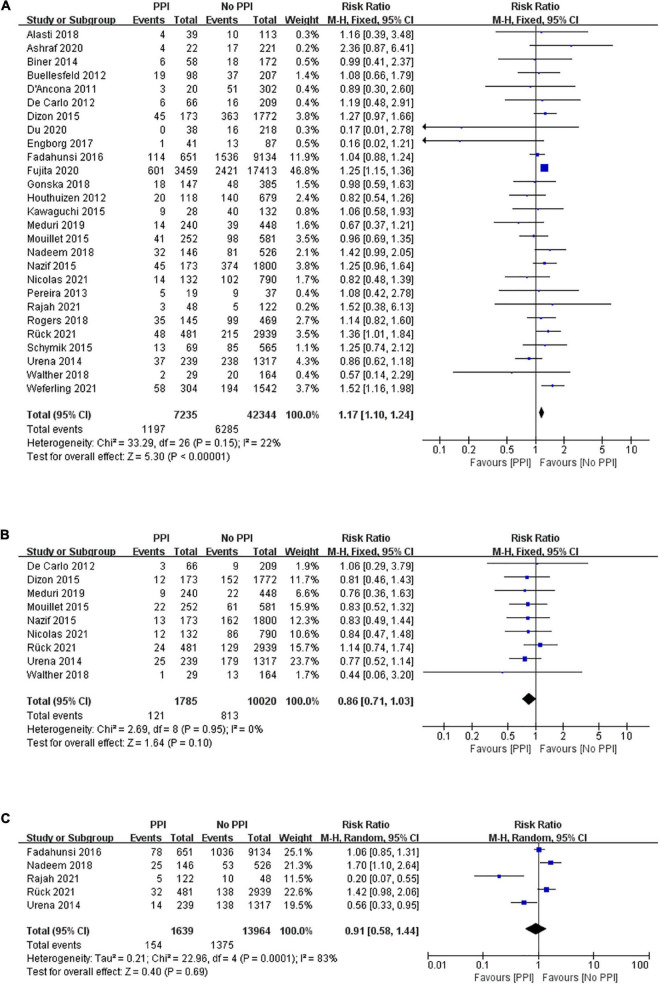
Forest plot of the risk of mid-term (1 year) **(A)** all-cause mortality, **(B)** cardiovascular mortality, and **(C)** heart failure rehospitalization in patients with post-procedural permanent pacemaker implantation (PPI) after transcatheter aortic valve replacement (TAVR).

The risk of mid-term all-cause mortality was pooled from 27 studies that included 49,579 patients, and 7,235 patients were implanted with permanent pacemakers after TAVR. There were 1,197 of 7,235 (16.54%) cases of all-cause mortality in the PPI group while 6,285 of 42,344 (14.84%) cases in the no PPI group. The pooled results demonstrated that patients with PPI had a higher risk of death than those without PPI following TAVR (RR, 1.17; 95% CI, 1.10–1.24; *P* < 0.00001; *I*^2^ = 22%; Figure 2A). After pooling the results from nine studies, no significant difference in mid-term cardiovascular death was observed (RR, 0.86; 95% CI, 0.71–1.03; *P* = 0.10; *I*^2^ = 0%; Figure 2B). The risk of 1-year heart failure rehospitalization was assessed in five studies using a random-effects model. As shown in Figure 2C, no significant difference was observed in heart failure rehospitalization (RR, 0.91; 95% CI, 0.58–1.44; *P* = 0.69; *I*^2^ = 83%).

### Long-Term (> 1 Year) Clinical Effects of Post-procedural Permanent Pacemaker Implantation After Transcatheter Aortic Valve Replacement

**FIGURE 3:**
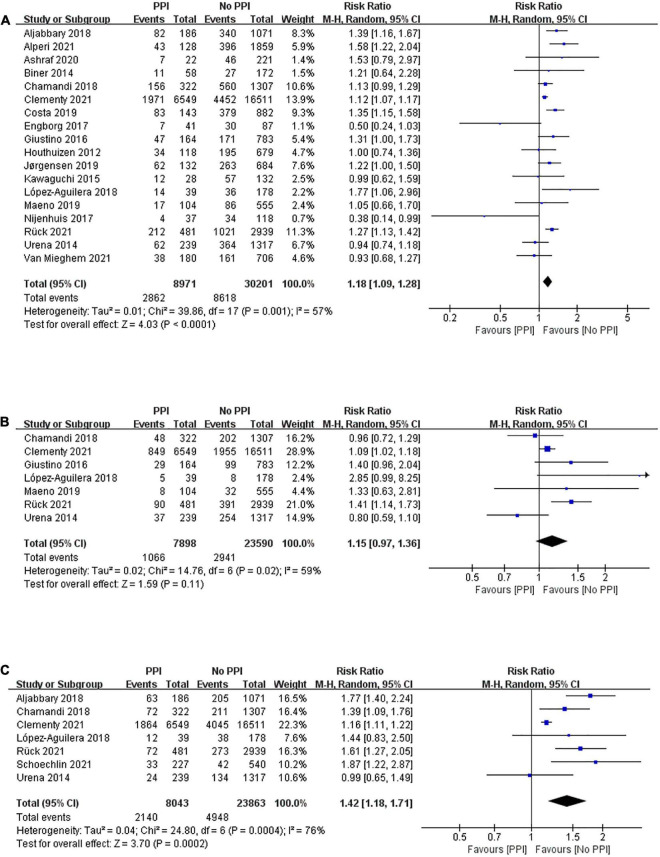
Forest plot of the risk of long-term (> 1 year) **(A)** all-cause mortality, **(B)** cardiovascular mortality, and **(C)** heart failure rehospitalization in patients with post-procedural PPI after TAVR.

Long-term mortality between patients with and without PPI after TAVR was reported in 18 studies enrolling 39,172 patients with a mean follow-up period of 2.59 years. A random-effects model was applied, and patients with PPI after TAVR had a higher risk of all-cause mortality than those without PPI after TAVR (RR, 1.18; 95% CI, 1.09–1.28; *P* < 0.0001; *I*^2^ = 57%; Figure 3A). However, there was no statistical difference in long-term risk of cardiovascular mortality between the two groups (RR, 1.15; 95% CI, 0.97–1.36; *P* = 0.11; *I*^2^ = 59%; Figure 3B) after a mean follow-up of 2.12 years. Seven studies demonstrated a deleterious effect of PPI on heart failure rehospitalization after a mean follow-up of 2.16 years (RR, 1.42; 95% CI, 1.18–1.71; *P* = 0.0002; *I*^2^ = 76%; Figure 3C).

### Echocardiographic Effects of Post-procedural Permanent Pacemaker Implantation After Transcatheter Aortic Valve Replacement

**FIGURE 4:**
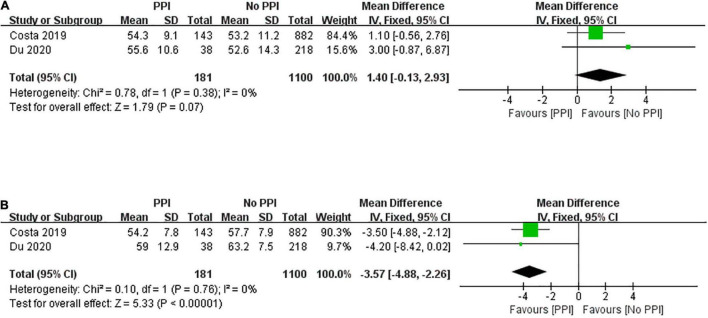
Forest plot of left ventricular ejection fraction at baseline **(A)** and 1-year follow-up **(B)** between patients with and without post-procedural PPI after TAVR.

Two studies reported LVEF both at baseline and 1-year follow-up. Figure 4A shows no significant difference in LVEF between the two groups at baseline (mean difference, 1.40; 95% CI, –0.13 to 2.93; *P* = 0.07; *I*^2^ = 0%). LVEF at 1-year follow-up after TAVR was assessed using a fixed-effect model, and the overall value of LVEF was significantly greater in the no PPI group than in the PPI group (mean difference, –3.57; 95% CI, –4.88 to –2.26; *P* < 0.00001; *I*^2^ = 0%; Figure 4B).

## Discussion

The results of this meta-analysis can be summarized as follows: (1) patients with post-procedural PPI show a higher risk of all-cause mortality at mid-term follow-up after TAVR; (2) post-TAVR PPI is associated with an increased risk of all-cause mortality and heart failure rehospitalization at long-term follow-up; and (3) post-procedural PPI adversely affect LVEF recovery on patients undergoing TAVR.

Twenty years after the first procedure in 2002 (55), TAVR has become a first-line treatment for patients with symptomatic severe aortic stenosis regardless of the estimated surgical risk (1–4). Although TAVR technology has matured significantly over the years, conduction abnormalities remain one of the major complications to be resolved. Currently, there is insufficient evidence to support that the newer-generation devices could reduce the rate of post-procedural PPI (56, 57). The underlying pathophysiological mechanisms compose of direct trauma, hemorrhage, inflammation, and ischemic injury of the conduction system during the expansion of the valve prosthesis (5). With accumulating TAVR cases, it is important to investigate the mid- to long-term clinical and echocardiographic outcomes of post-procedural PPI after TAVR.

Numerous studies have confirmed that RVP can negatively impact left ventricular function and increase the risk of the occurrence of atrial fibrillation (10, 58–60). The detrimental effects of RVP may elevate the risk of mortality and heart failure rehospitalization. As shown in our study, the pooled results revealed that patients undergoing PPI after TAVR had a higher risk of death at both mid- and long-term follow-up. They were also more likely to be hospitalized for heart failure during long-term follow-up. Similarly, a recent study containing the largest sample size reported that PPI after TAVR was independently associated with higher mortality and heart failure rehospitalization rate during follow-up, which was based on the entire France nationwide-level population (20).

We observed no significant difference in cardiovascular mortality and 1-year risk of heart failure rehospitalization between the two groups in our meta-analysis. The potential protective effects of PPI with respect to lethal bradyarrhythmias may counterbalance the negative effects of ventricular pacing. After the improvement of aortic stenosis, hemodynamic improvement of left ventricular function may compensate for the potential deleterious effects of ventricular pacing in such patients. In addition, implanting biventricular pacemakers in patients after TAVR may partially offset adverse effects linked to RVP.

Inconsistent with our results, few previous meta-analyses showed significant impacts of PPI after TAVR on clinical outcomes (13, 61, 62), except for a study by Faroux et al. (14), which was the first meta-analysis to reveal a significantly higher risk of all-cause death and heart failure rehospitalization in patients with PPI post-TAVR at 1-year follow-up. There are several explanations underlying the conflicting results in different studies. The small number of samples and short follow-up time may account for the distinct results. The occurrence and severity of pacing-induced cardiomyopathy are associated with ventricular pacing burden and duration, especially in patients with long-term pacing percentage ≥ 40% (11, 63, 64). Studies on TAVR have shown that new-onset conduction disturbances after TAVR may recover during follow-up, and about half of the patients requiring post-TAVR PPI are not pacing-dependent eventually (65–67). This may also partly explain why there was no significant difference in 1-year heart failure rehospitalization rates between the two groups.

Conduction disturbances occur commonly after TAVR, and an expert consensus algorithm was provided for managing post-TAVR conduction disturbances, but the optimal management of this complication is still unknown (68, 69). Schoechlin et al. (18) compared patients’ outcomes between different PPI implantation indications and revealed that the restrictive PPI strategy they adopted reduces the PPI rate significantly and is safe after a follow-up of 3 years. In consideration of the mid- to long-term negative effects demonstrated in our meta-analysis, we recommended adopting a relatively restrictive PPI strategy after TAVR, but the detailed indications and management need to be further explored. Furthermore, His-Purkinje system pacing (HPSP) allows for electrical stimulation signaling through the physiological conduction system, which has the potential to prevent pacing-induced dyssynchrony, heart failure hospitalization, and mortality (70–73). Previous studies have confirmed the feasibility and safety of HPSP in patients after TAVR. De Pooter et al. (74) found that the valve prosthesis can serve as an anatomical landmark for the implantation of the His-bundle lead. A multicenter study by Vijayaraman et al. (75) revealed that left bundle branch pacing had a higher success rate than His-bundle pacing after TAVR, with more ideal pacing parameters. Eleven patients with reduced left ventricular function who underwent HPSP successfully in this study showed significant LVEF improvement from 35 to 42% during follow-up. However, there is no systematic large-scale study evaluating the clinical and echocardiographic effects of HPSP in patients undergoing TAVR. Therefore, further studies are needed to focus on this area.

### Limitations

Several limitations of our meta-analysis should be acknowledged. First, most studies included in our meta-analysis were retrospective observational studies. Thus, prospective, multi-center, randomized comparative studies are urgently needed. Second, TAVR technology has developed over time, and the types of valve prostheses are different. Patients included in the prior studies might have different PPI inclusion criteria compared with later ones so the heterogeneity among studies was relatively high in our study. Third, we had inadequate numbers of studies reporting ventricular pacing percentage to assess any significance of pacing-induced cardiomyopathy. We also do not have enough information to study other complications of PPI, such as infection, pneumothorax, and pocket hematoma, which may result in significant clinical consequences outside of mortality. Last but not least, our study is a meta-analysis, and we lack access to individual patient data which may provide more information.

## Conclusion

The present meta-analysis provides evidence that post-TAVR PPI has negative clinical and echocardiographic effects at mid- to long-term follow-up. This study highlights the importance of identifying patients at high risk of developing conduction disturbances and requiring PPI after TAVR. Cardiologists should optimize treatment strategies and management of these patients. TAVR technology should also improve to reduce the incidence of such complications.

## Data Availability Statement

The original contributions presented in this study are included in the article/Supplementary Material, further inquiries can be directed to the corresponding author/s.

## Author Contributions

SX, EZ, JS, and JZ contributed to the conception and designed the study. SX, EZ, and JS extracted the data and evaluated the quality. SX and EZ analyzed the data and wrote the manuscript. ZQ, FZ, YW, XH, and JZ critically reviewed and revised the manuscript. All authors have read and approved the final version of the manuscript.

## Conflict of Interest

The authors declare that the research was conducted in the absence of any commercial or financial relationships that could be construed as a potential conflict of interest.

## Publisher’s Note

All claims expressed in this article are solely those of the authors and do not necessarily represent those of their affiliated organizations, or those of the publisher, the editors and the reviewers. Any product that may be evaluated in this article, or claim that may be made by its manufacturer, is not guaranteed or endorsed by the publisher.

## Abbreviations

TAVR: transcatheter aortic valve replacement
PPI: permanent pacemaker implantation
RVP: right ventricular pacing
LVEF: left ventricular ejection fraction
NOS: Newcastle-Ottawa Scale
RR: relative risk
CI: confidence intervals
HPSP: His-Purkinje system pacing.
