# Supplementary material for: Mid- to Long-Term Clinical and Echocardiographic Effects of Post-procedural Permanent Pacemaker Implantation After Transcatheter Aortic Valve Replacement: A Systematic Review and Meta-Analysis
Source: Front Cardiovasc Med. 2022 Jun 28;9:911234. doi: 10.3389/fcvm.2022.911234 (PMC9275565; doi:10.3389/fcvm.2022.911234)
Supplement: Supplementary file 1 [file Table_1.pdf]

Supplementary Table 1. Search strategies in Embase, Web of Science, and Cochrane Library.

Embase

Search	Query	Records
#1	Transcatheter Aortic Valve Replacement	17,866
#2	'Transcatheter Aortic Valve Replacement':ab,ti OR 'Transcatheter Aortic Valve Implantation':ab,ti OR 'TAVR':ab,ti OR 'TAVI':ab,ti	23,829
#3	#1 OR #2	27,496
#4	Cardiac Pacing, Artificial	6,540
#5	'pacing':ab,ti OR 'pace':ab,ti	77,118
#6	#4 OR #5	78,415
#7	Pacemaker, Artificial	39,079
#8	'pacemaker':ab,ti	51,547
#9	#7 OR #8	72,282
#10	#6 OR #9	133,845
#11	#3 AND #10	4,782

Web of Science

Search	Query	Records
#1	TS=(Transcatheter Aortic Valve Replacement OR Transcatheter Aortic Valve Replacement OR Transcatheter Aortic Valve Implantation OR TAVR OR TAVI)	22,670
#2	TS=(Cardiac Pacing, Artificial OR pacing OR pace)	352,418
#3	TS=(Pacemaker, Artificial OR pacemaker)	67,517
#4	(#2) OR #3	400,157
#5	(#1) AND #4	3,053

Cochrane Library

Search	Query	Records
#1	Transcatheter Aortic Valve Replacement	757
#2	(Transcatheter Aortic Valve Replacement):ab,ti,kw OR (Transcatheter Aortic Valve Implantation):ab,ti,kw OR (TAVR):ab,ti,kw OR (TAVl):ab,ti,kw	1102
#3	#1 OR #2	1110
#4	Cardiac Pacing, Artificial	1254
#5	(pacing):ab,ti,kw OR (pace):ab,ti,kw	6308
#6	#4 OR #5	6321
#7	Pacemaker, Artificial	1231
#8	(pacemaker):ab,ti,kw	2837
#9	#7 OR #8	2854
#10	#6 OR #9	7864
#11	#3 AND #10	175
